# Characterisation of *Clostridium difficile* Hospital Ward–Based Transmission Using Extensive Epidemiological Data and Molecular Typing

**DOI:** 10.1371/journal.pmed.1001172

**Published:** 2012-02-07

**Authors:** A. Sarah Walker, David W. Eyre, David H. Wyllie, Kate E. Dingle, Rosalind M. Harding, Lily O'Connor, David Griffiths, Ali Vaughan, John Finney, Mark H. Wilcox, Derrick W. Crook, Tim E. A. Peto

**Affiliations:** 1 National Institute for Health Research Oxford Biomedical Research Centre, John Radcliffe Hospital, Oxford, United Kingdom; 2 Medical Research Council Clinical Trials Unit, London, United Kingdom; 3 Nuffield Department of Clinical Laboratory Sciences, Oxford University, John Radcliffe Hospital, Oxford, United Kingdom; 4 Department of Zoology, Oxford University, Oxford, United Kingdom; 5 Nuffield Department of Clinical Medicine, Oxford University, John Radcliffe Hospital, Oxford, United Kingdom; 6 Department of Microbiology, The General Infirmary, Old Medical School, Leeds, United Kingdom; 7 Leeds Institute of Molecular Medicine, University of Leeds, Leeds, United Kingdom; Brown University School of Medicine, United States of America

## Abstract

**Background:**

*Clostridium difficile* infection (CDI) is a leading cause of antibiotic-associated diarrhoea and is endemic in hospitals, hindering the identification of sources and routes of transmission based on shared time and space alone. This may compromise rational control despite costly prevention strategies. This study aimed to investigate ward-based transmission of *C. difficile*, by subdividing outbreaks into distinct lineages defined by multi-locus sequence typing (MLST).

**Methods and Findings:**

All *C. difficile* toxin enzyme*-*immunoassay*-*positive and culture-positive samples over 2.5 y from a geographically defined population of ∼600,000 persons underwent MLST. Sequence types (STs) were combined with admission and ward movement data from an integrated comprehensive healthcare system incorporating three hospitals (1,700 beds) providing all acute care for the defined geographical population. Networks of cases and potential transmission events were constructed for each ST. Potential infection sources for each case and transmission timescales were defined by prior ward-based contact with other cases sharing the same ST. From 1 September 2007 to 31 March 2010, there were means of 102 tests and 9.4 CDIs per 10,000 overnight stays in inpatients, and 238 tests and 15.7 CDIs per month in outpatients/primary care. In total, 1,276 *C. difficile* isolates of 69 STs were studied. From MLST, no more than 25% of cases could be linked to a potential ward-based inpatient source, ranging from 37% in renal/transplant, 29% in haematology/oncology, and 28% in acute/elderly medicine to 6% in specialist surgery. Most of the putative transmissions identified occurred shortly (≤1 wk) after the onset of symptoms (141/218, 65%), with few >8 wk (21/218, 10%). Most incubation periods were ≤4 wk (132/218, 61%), with few >12 wk (28/218, 13%). Allowing for persistent ward contamination following ward discharge of a CDI case did not increase the proportion of linked cases after allowing for random meeting of matched controls.

**Conclusions:**

In an endemic setting with well-implemented infection control measures, ward-based contact with symptomatic enzyme-immunoassay-positive patients cannot account for most new CDI cases.

*Please see later in the article for the Editors' Summary*

## Abstract 2

A population-based study in Oxfordshire (UK) hospitals by Sarah Walker and colleagues finds that in an endemic setting with good infection control, ward-based contact cannot account for most new cases of *Clostridium difficile* infection.

## Editors' Summary

**Background:**

Hospital-acquired infections are common and occur when patients are infected with an organism found in the hospital or health-care environment. Hospital-acquired infections can sometimes cause no symptoms but often lead to illness or even death. A leading hospital-acquired infection is with the anaerobic bacterium *Clostridium difficile,* which causes gastrointestinal problems, including diarrhea, leading to severe illness and even death, especially in older patients or patients who are already seriously ill. Between 7% and 26% of elderly adult inpatients in hospitals may be asymptomatic carriers of *C. difficile,* and the spores that are formed by this organism can live outside of the human body for long periods of time and are notoriously resistant to most routine surface-cleaning methods. Following major hospital-associated outbreaks around the world, *C. difficile* infection has become a prime target for expensive prevention and infection control strategies.

**Why Was This Study Done?:**

Prevention strategies and infection control measures have contributed to reducing the incidence of *C. difficile* infection, however, to date, there have not been any robust evaluations of the impact of such strategies in reducing the spread of infection at the individual level. In order to implement improved, cost-effective policies, and to work out how to reduce incidence even further, a better understanding of person-to-person spread is crucial, especially as infection with *C. difficile* depends on a combination of factors, such as antibiotic exposure and host susceptibility. Therefore, the researchers conducted this study to examine in detail the transmission of *C. difficile* in hospital wards in order to give more insight and information on the nature of person-to-person spread.

**What Did the Researchers Do and Find?:**

The researchers used a population-based study in Oxfordshire, UK, to investigate hospital ward–based transmission of defined *C. difficile* strains from symptomatic patients by identifying *C. difficile* infection from routine clinical microbiological samples from 1 September 2007 to 31 March 2010. Throughout this period, Oxfordshire hospitals operated a rigorous infection control policy monitored by infection control staff, in which stool samples for *C. difficile* testing were taken from admitted patients with persistent diarrhea, and from patients with any diarrhea who were 65 years or older. The researchers tested all stool samples for *C. difficile* toxins by enzyme immunoassay, cultured positive samples, and genotyped *C. difficile* isolates by using multi-locus sequence typing (to identify strains, that is, sequence types), and finally, constructed networks of cases and potential transmissions (by tracing contacts for up to 26 weeks) for each sequence type identified.

In order to show which ward-based contacts potentially incorporated direct person-to-person spread and indirect transmission via the environment during shared ward exposure, the researchers analysed links (ward contacts) between the first case (the donor) and the second case (the recipient) for all pairs of cases with the same sequence type. The researchers then calculated the minimum infectious period by measuring the time between the first infected stool sample from the donor and ward contact with the recipient, and calculated the incubation period as the time between this ward contact and the first infected stool sample in the recipient. To reduce the possibility of shared ward contacts occurring by chance, the researchers used patients with negative enzyme immunoassay stool samples as controls to estimate how often such ward contacts reflected actual transmission rather than chance.

Over the study period, almost 30,000 stool samples from almost 15,000 patients were tested for *C. difficile*, with 4.4% (1,282) found positive for *C. difficile* in enzyme immunoassay and culture. With genotyping, the researchers identified 69 strains (sequence types) of *C. difficile*. The researchers found that the majority (66%) of cases of *C. difficile* infection were not linked to known cases, and only 23% had a credible ward-based donor sharing the same sequence type of *C. difficile*. Furthermore, the researchers found that most probable transmissions occurred less than one week after the onset of symptoms, with a minority (10%) occurring after eight weeks. Most incubation periods were less than four weeks, but a few (13%) were more than 12 weeks. Importantly, even after allowing for the random meeting of matched controls and for persistent ward contamination, the proportion of linked cases did not increase following ward discharge of a *C. difficile* infection case.

**What Do These Findings Mean?:**

These findings show that in an endemic setting with well-implemented infection control measures, ward-based contact with symptomatic, enzyme-immunoassay-positive patients cannot account for most new cases of *C. difficile* infection. Crucially, these findings mean that *C. difficile* infection might not be effectively controlled by current strategies to prevent person-to-person spread. Although the researchers were able to distinguish different strains of *C. difficile*, there were insufficient numbers of these different strains to deduce whether the results they obtained might be different if there was a different combination of strain types, that is, if some strains were spreading more in hospitals than others. Finally, in order to determine what other types of control interventions are required to reduce the spread of *C. difficile*, a better understanding of other routes of transmission and reservoirs of infectivity is needed.

**Additional Information:**

Please access these web sites via the online version of this summary at http://dx.doi.org/10.1371/journal.pmed.1001172.

- This study is further discussed in a *PLoS Medicine* Perspective by Stephan Harbarth and Matthew Samore
- The US Centers for Disease Control and Prevention provides information about *C. difficile* infection, as does the UK Health Protection Agency
- The World Health Organization has published a guide for preventing hospital-acquired infections

## Introduction

Infection with *Clostridium difficile* is a leading cause of healthcare-associated diarrhoea, which is almost exclusively precipitated by antibiotic exposure that disturbs the normal intestinal flora, allowing *C. difficile* to flourish [1]. Following major hospital outbreaks that resulted in high morbidity/mortality [2],[3], *C. difficile* infection (CDI) has become the focus of multi-faceted and costly prevention strategies [1],[4].

Rigorous implementation of these infection control measures is believed to have reduced disease incidence [5],[6]; however, robust evaluation of the impact of these control measures on person-to-person spread is largely lacking. Further, the infectious and incubation periods of CDI are less well defined than for many pathogens [1] for which much data illuminating transmission arises from point-source outbreaks (e.g., norovirus [7], salmonella [8], and HIV [9]) or challenge studies (e.g., typhoid [10], syphilis [11], and malaria [12]). In contrast, CDI generally requires a combination of pathogen acquisition, antibiotic exposure, and host susceptibility [13], and so transmissions that do not immediately result in disease can go undetected. A better understanding of person-to-person spread is critical for promoting rational and cost-effective control policies.

Here we study endemic CDI in a large geographical region over a 2.5-y period, where a recent genotyping scheme [14] has revealed many distinct lineages, with each lineage containing a small enough number of cases to analyse the lineage as a separate outbreak. Our objective was to investigate ward-based transmission of defined *C. difficile* strains from symptomatic cases, the interval between CDI diagnosis and putative onward transmission, incubation periods, and the proportion of cases arising from ward-based transmission from known symptomatic CDI cases, in order to identify and/or better target infection control measures.

## Methods

### Ethics Statement

This was a pre-specified analysis within the Infections in Oxfordshire Research Database, an anonymized linked electronic database approved by the Oxford Research Ethics Committee (09/H0606/85) and the National Information Governance Board (5-07(a)/2009).

### Setting

The Oxford Radcliffe Hospitals (ORH) NHS Trust (1,700 beds) provides >90% of hospital care, and all acute services, to ∼600,000 people residing in Oxfordshire, United Kingdom. The Trust has two large sites in Oxford—the Churchill (medical specialities, cancer centre; two floors) and John Radcliffe (acute services, surgical specialities, women's centre, children's centre; seven floors)—and a smaller district hospital 35 miles north of Oxford (two floors). All ORH hospitals have discrete wards containing 20–30 beds in 4–6 bedded bays with shared bathrooms and only 2–4 side rooms: only a few specific wards (notably elderly medicine) are all single rooms. The ORH microbiology laboratory tests all stool samples from the region, including from other healthcare facilities (smaller mental health and specialist orthopaedic trusts, and community hospitals) and general practitioners (primary care). This population-based study included all CDI cases identified from routine clinical samples at the ORH microbiology laboratory from 1 September 2007 to 31 March 2010.

Throughout this period, the ORH hospitals operated a rigorous infection control policy (Table A in Text S1), which required samples to be sent for *C. difficile* testing from any admitted patient with diarrhoea (locally defined as ≥3 unformed stools, i.e., taking the shape of the container, within 24 h), and for oral vancomycin treatment to be initiated as first-line empiric therapy. Compliance was monitored weekly by infection control staff, with immediate feedback (Table A in Text S1). UK Department of Health policy also required that all unformed stool samples from those aged ≥65 y were sent to the laboratory be tested for *C. difficile*, whether or not the patient met the above diarrhoea criteria and *C. difficile* testing had been requested by the clinician sending the sample. All samples were tested by enzyme immunoassay (EIA) for *C. difficile* toxins A and B (Meridian Bioscience). EIA-positive samples were cultured [14] and *C. difficile* isolates genotyped by multi-locus sequence typing (MLST) [14]. A single colony from each sample was typed, except for morphologically distinct colonies, which were typed separately. EIA-positive, culture-negative isolates were not considered in analysis; because culture is one of two gold standards (the other being cell cytotoxicity [15]) and false EIA-positive rates up to 20% are well recognised [16], these most likely represent false positives.

### Analysis

**Figure 1:**
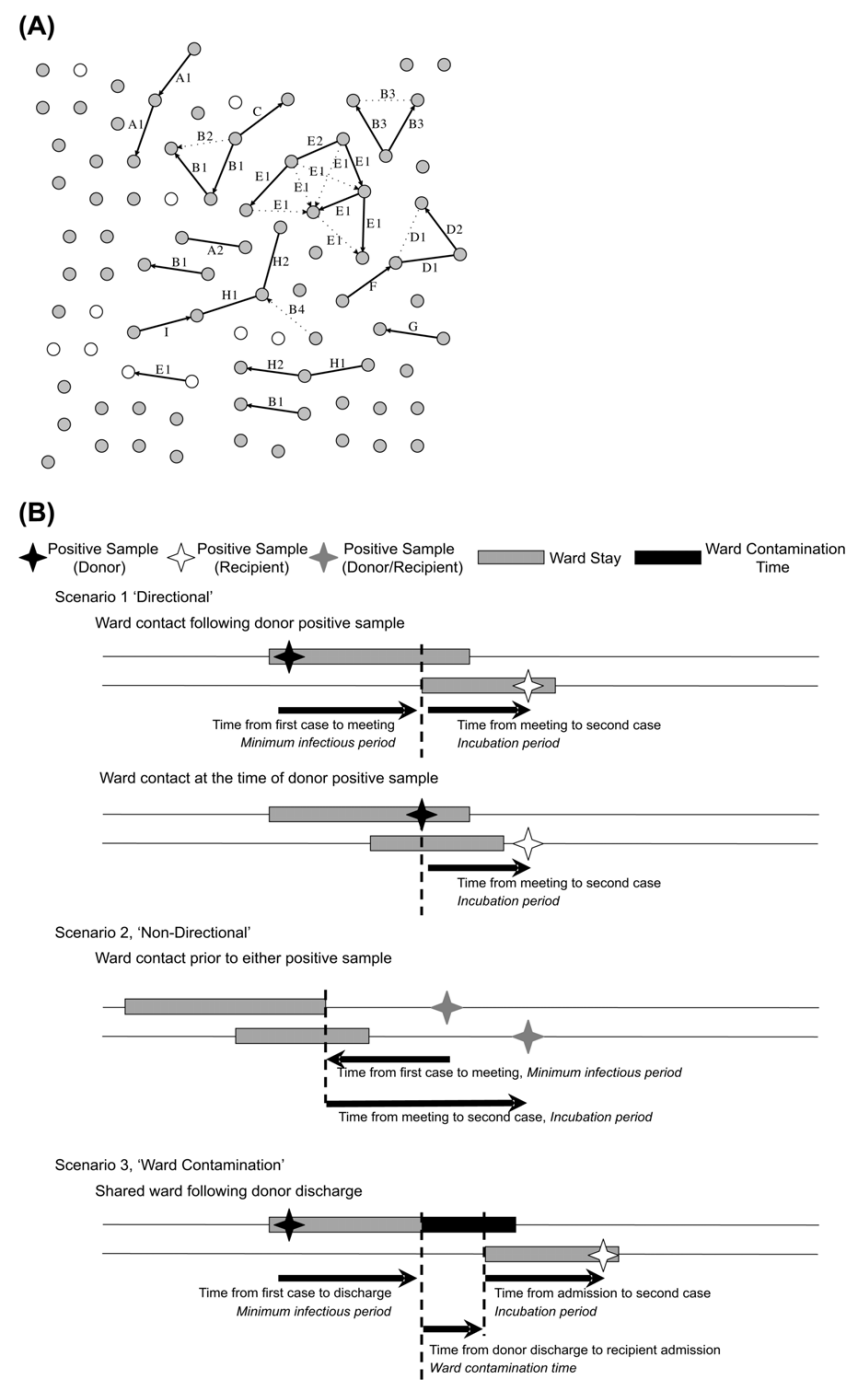
Ward-based links. (A) Ward-based links between cases of an example ST, ST-2. Cases from the first part of the study, 1 September 2007–29 February 2008 (the run-in period, where the source of the cases may plausibly be before 1 September 2007), are shown as white circles, and cases from the remainder of the study, 1 March 2008–31 March 2010 (the test period) are shown in grey. Where patients with CDI shared time on a common ward before either or both patients' CDI, links are indicated with solid or broken lines, representing possible transmission events for maximum allowable infectious and incubation periods of 8 and 12 wk, respectively. All potential ward-based transmission links are included; the single most plausible link to each recipient based on minimising infectious period is shown in solid lines. Links arising from ward contamination persisting after discharge of the donor patients are not shown. Letters indicate wards on which contact events occurred; wards that are related by specialty have the same letter but different numbers. Links of known direction (ward contact after potential donor CDI and before recipient CDI) are indicated with an arrowhead. Cases are approximately evenly spaced, with linked cases adjacent for the purpose of illustration (i.e., the distance between circles does not generally correspond to any physical quantity). (B) Schema showing different types of ward-based contact.

The first EIA-positive, culture-positive *C. difficile* sample of each sequence type (ST) from each patient was included in the primary analysis (55/927 [6%] patients had multiple CDI with different STs). A network of ward-based contacts between cases, representing potential transmission events, was created for each ST (Figure 1A). Links were made when two CDI cases shared time on a ward, either (i) after the first case's sample (the “donor”) and before the second case's sample (the “recipient”) (directional links), or (ii) before both cases' samples were taken (non-directional links) (Figure 1B). As defined, ward-based contacts incorporated direct person-to-person spread and indirect transmission via the environment during shared ward exposure. For each link, we defined the putative “minimum infectious period” as the time between the first sample from the potential donor and ward contact with the recipient. We defined the putative “incubation period” as the time between this ward contact and the first sample in the recipient. For a detailed description see Text S1. To avoid double-counting when multiple possible donors had ward-based contacts with the same recipient, we present the characteristics of the most plausible transmission link, chosen assuming that donors were most infectious closest to diagnosis (i.e., minimising the putative infectious period) (see Figure B in Text S1 for results for all links).

Two separate sets of networks were constructed. In the first, contacts up to 26 wk from either donor or recipient—longer than the currently accepted infectious and incubation periods [1]—were allowed to inform the most likely infectious/incubation periods. In the second set, incubation/infectious periods were restricted based on results from the first set of networks, and we included the possibility of transmission from the ward environment after the donor was discharged. We defined the potential ward contamination time as the time between donor discharge from and recipient admission to the ward, allowing up to 26 wk, and assuming direct ward contact was a more likely route of transmission than ward contamination. The proportion of cases that could be explained by credible ward-based transmission from symptomatic EIA-positive, culture-positive patients was calculated only for CDIs from 1 March 2008 to 31 March 2010, the “test” cases, to allow for new CDIs resulting from acquisition some time previously. However, all samples during and after a 6-mo “run-in” period, 1 September 2007–29 February 2008, were considered as possible donors to “test” cases.

### Sensitivity Analysis

One key assumption is choosing the most plausible potential transmission link based on minimising infectious periods; analyses were therefore repeated with the alternative assumption of minimising incubation periods. Some patients had successive EIA-positive samples with the same ST. Assuming these represent on-going diarrhoea due to the same infection, in a further sensitivity analysis donor infectious periods were calculated relative to their most recent (rather than their first) EIA-positive sample with this ST prior to the ward contact. To estimate the effect of missing cases, not detected as part of routine testing, the analysis was repeated after randomly removing cases from the complete dataset. Networks were regenerated and the proportion of the remaining cases with a credible ward-based donor calculated. Between 5% and 50% of the original cases were removed, with 1,000 repetitions undertaken for each 5% increment. Extrapolation of the results was used to estimate the possible effect of missing cases.

### Controls

To calculate the proportion of links between cases that might be the result of shared ward contacts occurring by chance rather than actual transmission, ward-based networks were constructed for the same number of patients without CDI for each ST, randomly sampled 1,000 times. The main controls were patients with EIA-negative diarrhoea. The proportion of controls that could be linked by shared time and hospital space was used to adjust the number of linked CDI cases for chance meetings merely due to movements around the hospital, and to therefore provide an estimate of the proportion of cases linked by actual transmission. (See Text S1 for other controls.)

### Statistics and Computation

Graphs and standard statistics were done using STATA 11.1. Networks were drawn using NetworkX and Python Graphviz libraries. Random samples were generated and networks analysed using MySQL 5.1.

## Results

### Samples and STs

**Figure 2:**
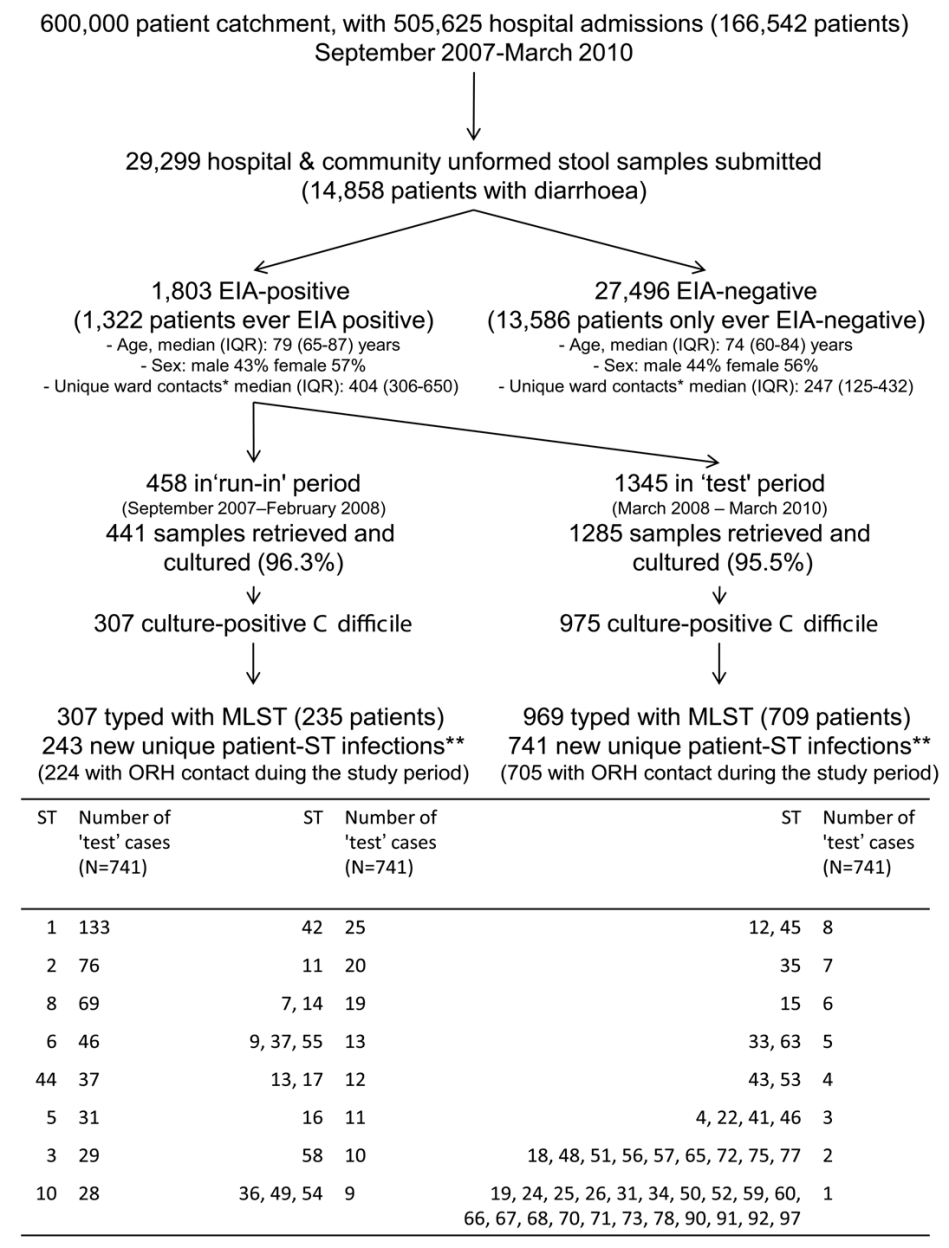
Samples and patients, 1 September 2007–31 March 2010. * The number of unique patients with whom the index patient shared a ward during the study period. ** 55 patients had multiple ST infections during the study period: 54 patients had two STs, and one had four STs. Six isolates were not successfully typed (either because of ambiguous sequences despite repeated testing or because of loss of sample). Note: 55 cases had no record of ORH inpatient admission during the study. Some patients had different ST infections in the test and run-in periods and are therefore counted in both.

From 1 September 2007 to 31 March 2010, 29,299 unformed stool samples from 14,858 Oxfordshire patients were tested for *C. difficile*: 102 tests per 10,000 overnight stays among ORH inpatients and a mean of 30 and 208 samples per month from day cases/outpatients/emergency department and non-ORH locations, respectively (Figure A in Text S1). In total, 1,803 (6.2%) tests were EIA-positive, a rate of 9.4 CDIs/10,000 overnight stays among ORH inpatients and a mean of 2.8 and 12.9 CDIs/month from day cases/outpatients/emergency department and non-ORH locations, respectively (Figure A in Text S1), with relatively little variation over the study period. 1,282 (4.4%) tests were both EIA-positive and culture-positive, of which 1,276 (from 927 patients) were genotyped by MLST (Figure 2): 69 distinct STs were identified. 893 (70%) cases occurred in ORH inpatients, of which 456 (51%) were admitted under acute/elderly medicine, 152 (17%) general surgery, 68 (8%) renal/transplant, 52 (6%) haematology/oncology, 38 (4%) specialist surgery, 29 (3%) trauma/orthopaedics, and the remaining 98 (11%) under other smaller specialities.

**Figure 3:**
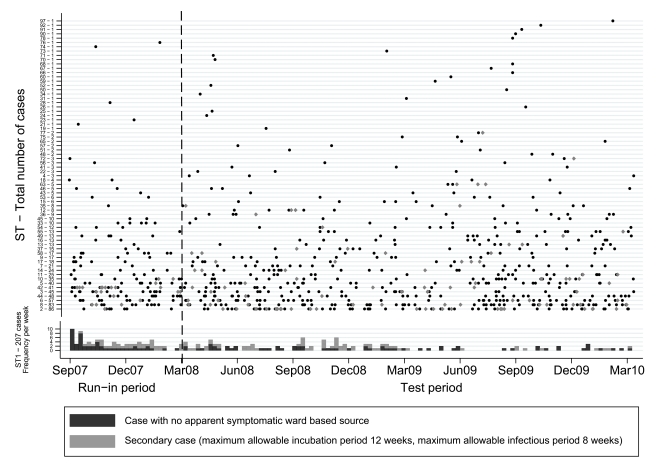
Time distribution of *C. difficile* cases, by sequence type. The first EIA-positive sample per ST per patient is plotted as a point for STs other than ST-1 (PCR-ribotype-027), where the 207 cases are instead shown as a histogram per week. Secondary cases are those with an identified credible donor based on incubation periods up to 12 wk and infectious periods up to 8 wk.

*C. difficile* isolates were grouped into a 6-mo run-in followed by a 2-y test period. Figure 3 shows when isolates of each ST occurred over time, and suggests both temporal clustering and a large number of sporadic cases. Figure 1A uses ST-2 (the most common non-PCR-ribotype-027 ST) to illustrate the ward-based links that could be made between same-ST cases. 55(6%) CDI cases had no ORH exposure during the study and were excluded from further analysis, since, by definition, no ward-based donor or onward transmission can be identified.

### Infectious/Incubation Periods

**Figure 4:**
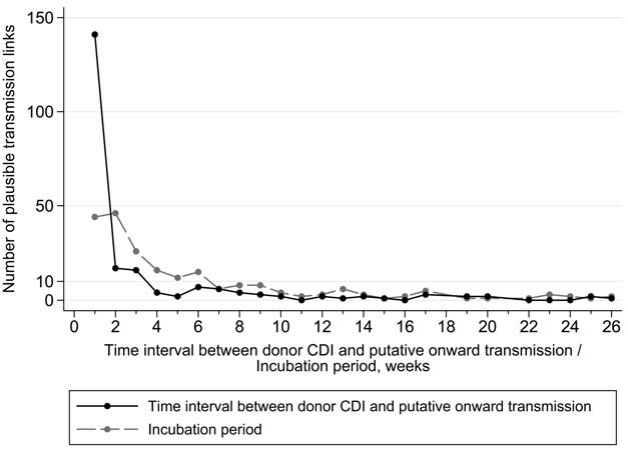
Distribution of infectious and incubation periods for putative transmissions within 69 STs and 705 “test” CDI cases. Only the most plausible transmission links, where the direction of transmission is known (ward contact after potential donor CDI and before recipient CDI), are plotted, assuming maximum allowable infection and incubation periods of 26 wk and no ward contamination persisting after donor discharge (see Figure B in Text S1 for distributions from all potential links). Times are plotted rounded up to the nearest week, e.g., intervals of 0–6 d are plotted as 1 wk.

To include all links likely to represent true transmission, ward contacts up to 26 wk from either case, longer than biologically expected [1], were initially allowed. Figure 4 shows the distribution of the minimum infectious/incubation periods from the 218 most plausible of the 362 potential transmission links where ward contact occurred between potential donor and recipient CDI (directional links). Figure B in Text S1 shows all possible links, with similar results. With 26-wk limits, of 705 test cases from 1 March 2008 to 31 March 2010, 408 (58%) were unlinked, 74 (10%) were donors of infection without an identified source for their own infection, and 223 (32%) had a credible ward-based donor sharing the same ST, assuming no ward contamination persisted after discharge (Table B in Text S1).

Not all ward-based links between cases represent true transmissions, since two patients can meet by chance without a transmission occurring. Consistent with this, the number of ward-based links with longer infectious/incubation periods approached a constant background rate after 8–12 wk. Most time intervals between potential donor CDI and putative onward transmission (minimum infectious period) were ≤1 wk after the donor was diagnosed (141/218, 65%), with the majority ≤4 wk (178/218, 82%), and few >8 wk (21/218, 10%) (Figure 4). The median (interquartile range) minimum infectious period for the most plausible directional potential transmission link was 1 (0–14) d, compared with 4 (0–20) d and 8 (0–33) d for the most plausible and all potential links, respectively. The most common incubation period (from ward-based contact with donor to recipient CDI) was ≤4 wk (132/218, 61%), with few >12 wk (28/218, 13%) after the ward-based contact. The median (interquartile range) incubation periods for the most plausible directional potential transmission links, the most plausible links, and all potential links were 18 (8–42) d, 24 (10–61) d, and 33 (13–74) d, respectively.

### Proportion of Cases with a Credible Donor

**Figure 5:**
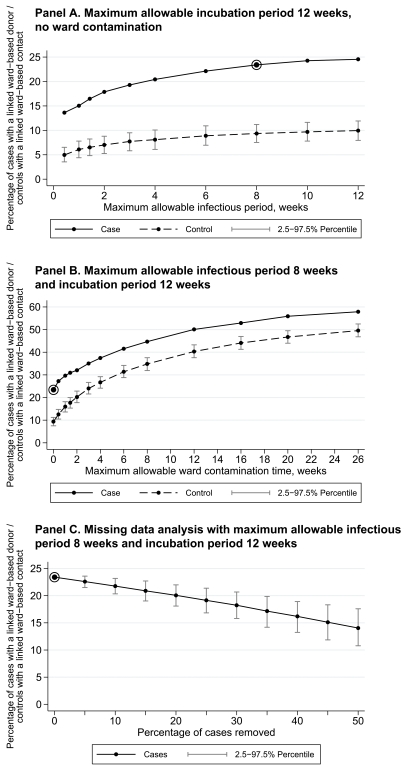
Percentage of cases explained by ward-based contact with a *C. difficile* toxin EIA-positive donor. The circled dots indicate a potential upper bound for the proportion of linked CDI cases. Controls are drawn at random 1,000 times from hospital-exposure-matched EIA-negative patients; linked ward-based contacts for controls therefore represent the proportion of ward-based contacts that would likely arise in the CDI cases merely from chance movements around the hospitals. Net linked cases correct the overall total linked CDI cases for these chance ward meetings estimated from controls. (A) Maximum allowable incubation period of 12 wk, no ward contamination. (B) Maximum allowable infectious period of 8 wk and incubation period of 12 wk. (C) Missing data analysis with maximum allowable infectious period of 8 wk and incubation period of 12 wk.

Figure 5A shows the proportion of CDI cases with a credible ward-based donor from final networks based on a 12-wk maximum incubation period with no ward contamination persisting beyond discharge. With an 8-wk maximum infectious period, 465 (66%) of the 705 test cases were unlinked, and 165 (23%) had a credible ward-based donor sharing the same ST (Table B in Text S1). The percentage of cases with a credible donor was highest in renal/transplant (37%), haematology/oncology (29%), and acute/elderly medicine (28%), with fewer linked cases in general surgery (20%), trauma/orthopaedics (16%), and other medical (13%) and surgical (6%) specialities. Increasing the maximum allowable infectious period to 12 wk increased the proportion with credible ward-based donors to ∼25%, whilst decreasing the maximum allowable incubation period to 4 wk decreased it to ∼17%. The proportion of EIA-negative controls, matched for exposure to other hospital patients, with ward-based links to other controls using the same algorithm was ∼10% (Figure 5A). These links provide an estimate of the proportion of links among the CDI cases that were likely to have arisen by chance meetings between patients as they moved around the hospitals, and suggest that a substantial minority of the possible transmission links seen in cases could actually be chance admissions to the same ward. After adjustment for chance meetings, a net 16% of cases are linked by probable transmission events. Varying other matching criteria, including diarrhoea-free controls, produced results similar to those in Figure 5.

### Ward Contamination beyond Discharge

Figure 5B shows the proportion of CDI cases with a credible ward-based donor based on an 8-wk maximum allowable infectious period and a 12-wk maximum allowable incubation period, allowing for transmission from a ward after donor discharge up to a maximum 26 wk. The numbers of links arising from putative ward contamination increase similarly in CDI cases and controls without CDI, giving few additional net linked cases due to ward contamination after CDI case discharge. This suggests that such links are more likely due to chance admissions of cases with the same ST to the same ward rather than transmission from persistent ward contamination.

### Sensitivity Analyses

Results were similar when (i) restricting links to those where the point of potential transmission was known more precisely (0- to 1-d ward contact durations only), (ii) minimising incubation rather than infectious periods, and (iii) calculating infectious/incubation periods relative to the most recent, rather than the first, donor sample (Table 1).

**Table:** **Table 1.** Summary of results and sensitivity analyses.

Summary	Description	Result/Impact
**Analyses**	Distribution of infectious and incubation periods	Most of the putative transmissions identified occurred shortly (≤1 wk) after the onset of symptoms (141/218, 65%), with few >8 wk (21/218, 10%)
		Most incubation periods were ≤4 wk (132/218, 61%), with few >12 wk (28/218, 13%)
	Proportion of cases with a credible ward-based symptomatic EIA-positive source	No more than 25% of cases could be linked to a symptomatic EIA-positive ward-based inpatient source
	Role of ward contamination after discharge of an infected case	Allowing for persistent ward contamination following ward discharge of a patient with CDI did not increase the proportion of linked cases after allowing for random meeting of matched controls
**Sensitivity analyses**	Alternative assumption: minimising incubation periods rather than minimising infectious periods	Similar results obtained for all analyses
	Alternative assumption: incubation and infectious periods calculated relative to most recent EIA-positive sample rather than first EIA-positive sample	
	Analysis restricted to cases where point of transmission known more precisely (≤1-d contacts)	
	Distribution of infectious and incubation periods compared including and excluding links to multiple possible donors (before and after pruning)	
	Choice of controls	Similar results for EIA-negative controls and diarrhoea-free controls matched for hospital exposure
	Estimation of the impact of missing data by removal of observed cases at random	The proportion of the remaining cases with a credible donor fell from 23% to 22% if 10% of cases were randomly removed, and to 18% if 30% of cases were removed. Extrapolation suggests that if 10%–50% of true CDI cases had been missed, the proportion of cases with a credible ward-based donor would increase to <40%

Investigation of the impact of missing data showed that, under plausible upper limits on the maximum allowable infectious/incubation periods, the proportion of the remaining cases with a credible donor fell from 23% to 22% if 10% of cases were randomly removed, and to 18% if 30% of cases were removed (Figure 5C). Extrapolation suggests that had the 4% of EIA-positive samples not retrieved for culture actually had STs obtained, the proportion of cases with a credible donor might have been ∼25%, rather than ∼23%.

## Discussion

Our uniquely large dataset of genotyped *C. difficile* isolates, ∼20% of which were the hypervirulent PCR-ribotype-027/NAP-1/BI/ST-1 strain, was obtained from patients in defined ward locations, in a group of hospitals from a large UK NHS Trust practising rigorous infection control. To our knowledge for the first time for symptomatic *C. difficile* EIA-positive, culture-positive cases, we have investigated (i) the proportion of cases attributable to credible ward-based transmission from other symptomatic cases, (ii) the time between CDI diagnosis and potential onward transmission (denoted the minimum infectious period) and between putative acquisition and CDI diagnosis (incubation period) based on these putative transmissions, and (iii) the possible impact on transmission of ward contamination persisting following discharge of CDI cases. Crucially, our findings are based on data collected over 2.5 y, and are thus less susceptible to biases inherent during CDI outbreaks. Our CDI rates are all within the 3.8–9.5/10,000 overnight stays typically observed in endemic settings [1], our infection control policy is based on widely available guidance [4], and our group of hospitals contains both specialist and general hospitals typical of those found in the UK. We therefore believe these findings should be generalisable to other endemic settings.

Plausible ward-based transmissions were most commonly observed during the week following the first EIA-positive sample. This supports the current recommendations for immediate isolation of symptomatic patients, and is consistent with evidence showing that *C. difficile* shedding is most prominent during episodes of diarrhoea [17]. Current guidance suggests isolation should continue until 48 h after diarrhoea resolution [4],[18]; our data show that the potential for transmission persisted for up to 8 wk. We found no evidence of substantial onward transmission from wards after patient discharge; whilst this clearly can occur, our data suggest the straightforward enhanced cleaning protocols used were sufficient to prevent most environment-related transmission occurring after CDI cases left wards. We could not distinguish between direct person-to-person spread and indirect (spore-based) transmission via the environment during shared ward exposure, classing both as “ward contact”.

Our data suggest that incubation periods most commonly last a few days to 4 wk, but extending these up to 12 wk can provide plausible ward-based transmission links. Previous studies have been inconclusive. Cohort studies taking serial samples from inpatients (likely receiving antibiotics) suggest a median 2–3 d from first positive test to symptoms [19]–[21]; epidemiological investigations showing that hospital exposure over the last 60 d is a risk factor for community-onset disease suggest that incubation periods may be considerably longer [22]. Providing exact limits is complicated by the additional dependence of symptom onset on antibiotic use, data not collected electronically in our hospitals and therefore not available for this study. As well as informing outbreak investigation, our data broadly support the current surveillance definitions in recent Society for Healthcare Epidemiology of America/Infectious Diseases Society of America guidelines [1], which define cases occurring 0–4 wk post-discharge as “healthcare associated” and 4–12 wk post-discharge as having a possible healthcare facility origin.

In our study no more than ∼25% of patients with detected symptomatic CDI could plausibly have acquired the infection from other patients with EIA-positive symptomatic CDI via ward contact. Chance meetings between patients, without transmission occurring, could mean that this 25% is in itself an overestimate. Similarly, in a study of ten nosocomial pathogens, not including *C difficile*, in an intensive care unit setting, investigators found that only 15% of infections could be attributed to ward-based patient-to-patient spread based on molecular typing [23]. As expected, the proportion of CDI cases with identifiable donors varied according to location/speciality, because of differences in risk of CDI acquisition by susceptible patients, such as those on renal wards. The fact that patients on our elderly medical wards are nursed in single rooms probably lowered the proportion of linked donors and recipients somewhat compared to cohort wards. The ∼25% of cases linked via ward contact represent a major hospital-acquired infection problem; however, the remaining ∼75% of unexplained transmission raises concern about other acquisition routes not captured by this study. These could include transmission from patients with falsely EIA-negative symptomatic CDI [16],[24], asymptomatic carriers [25] (e.g., patients, relatives, and staff), or significant acquisition within the community (e.g., from food [26] or animals [27]) with importation into the hospitals.

Since (i) 800–1,000 EIA tests were performed each month, (ii) there was an ORH policy of universal testing of nosocomial diarrhoea (Table A in Text S1), (iii) the same laboratory also processed all community samples, and (iv) all unformed stool samples from patients >65 y old were routinely tested for *C. difficile*, large numbers of completely untested inpatient or community symptomatic patients are unlikely. Our 6.2% *C. difficile* EIA positivity rate is similar to the average 6.45% for 170 English hospitals in 2008 [28], suggesting we were unlikely to be identifying many fewer EIA-positive cases than other typical UK hospitals. The particular EIA assay used in our laboratory has an independently reported sensitivity of 91.7% (84.7%–96.1%) [29]: this relatively low sensitivity (common to all toxin EIAs [16],[24]) makes it possible that ∼10% of cases could have been missed because of false-negative EIAs. However, because of widespread concerns about test sensitivity, for most of the study period (to 31 December 2009), multiple diarrhoeal samples were submitted from each patient, either simultaneously or serially, reducing the chance of completely missing patients with symptomatic CDI. Mixed CDIs could be another reason for missing cases, although it is plausible that the most prevalent cultured strain that was sequence typed would also be the one most likely to be transmitted. Relatively low rates of mixed infection (2%–13%) have been identified in previous investigations, most with limited sample sizes, that examined multiple *C. difficile* colonies from single faecal samples [30]–[34]. Extrapolation from Figure 5C suggests that if the transmission behaviour of ∼10%–20% missed cases were similar to that of the observed cases, the proportion of linked cases might be a few percentage points higher than 25%. In a more extreme situation, where 50% of cases had been missed, the proportion of linked cases would still be <40%. Unidentified symptomatic cases might account for more transmission than observed cases if they were more infectious, e.g., because of lack of isolation. However, multiple transmissions over a short period of time from an unidentified donor would result in ward-based clusters of cases, which within our analysis would result in recipients being falsely assigned as transmitting to each other. While the true donor would remain unidentified, our analysis would nevertheless classify any such transmissions as ward-based and thus would capture a large proportion of such outbreaks in the linked cases already reported. The high numbers of unlinked cases thus suggests that increased transmission from unidentified symptomatic cases is likely to be uncommon.

Asymptomatic carriage of *C. difficile*, whilst possibly protective against subsequent CDI in the carrier [35], may still be a source for transmission [25]. If there were undetected ward-based transmission from asymptomatic carriers, our results would underestimate the proportion of patients that had acquired CDI as inpatients, similarly to our analysis removing cases at random. However, as above, multiple transmissions from an unidentified asymptomatic donor would likely result in the false attribution of direct ward-based contacts between recipients, and the high numbers of unlinked cases argues against a significant proportion of infection arising from a few highly infectious unidentified carriers. However, as between 7% and 26% of adult inpatients may be asymptomatic carriers of *C. difficile* [1], even if these patients infect relatively few patients each, asymptomatic carriage may be an important route of transmission, and requires further investigation.

At the start of this study, and in common with the wider clinical community [1], we had expected that the majority of CDI cases would be attributable to in-hospital transmission from other known symptomatic cases. Whilst the limitations discussed above imply that total in-hospital transmission could be greater than in-hospital transmission from this group, nevertheless, our findings suggest that, contrary to prevailing beliefs, ward-based transmission from known EIA-positive symptomatic cases cannot account for the majority of new CDIs. These are the only cases currently visible to infection control teams, and therefore are the only cases directly targeted by current infection control policies and practice. Improved tests, such as those based on nucleic acid/PCR-based methods, might improve detection rates [36], and it should be noted that even infections arising from an unknown source are to a certain extent preventable by measures such as good antimicrobial stewardship [4].

The fact that we were able to divide 1,276 isolates into 69 distinct STs in the proportions given in Figure 2, with the number of cases of each ST small enough to analyse as separate lineages, suggests that MLST is reasonably discriminatory for identifying transmission. The study was not powered to analyse the transmission properties of each ST independently; therefore, more data are required to comment on the extent to which the findings may have varied with a different mix of prevalent strains. In future, whole genome sequence data promise to provide further insights; the isolates from this study are currently undergoing whole genome sequencing to examine the microevolution of transmitted strains of *C difficile*. Such data might refute the possibility of transmission between epidemiologically linked cases with the same ST that differ substantially at the whole genome level, thus reducing the probability that chance ward meetings of patients are misattributed as transmission events based on shared STs. Also, genetically identical but apparently epidemiologically unlinked cases may be identified, suggesting other routes of transmission. Our heuristic analyses provide a foundation for more sophisticated probabilistic models for *C. difficile* transmission, which could incorporate transmission from unobserved cases.

In summary, in this endemic setting with well-implemented infection control measures, up to three-quarters of new CDIs are not easily explained by conventional assumptions of ward-based transmission from symptomatic patients and so may not be targeted by current interventions. A better understanding of other routes of transmission and reservoirs is needed to determine what other types of control interventions are required to reduce the spread of *C. difficile.*

## Supporting Information

Text S1 **Additional methods and results.** Table A: Interventions in place at the start of the study (1 September 2007), and changes during the study (through 31 March 2010). Table B: Network characteristics. Figure A: *C. difficile* tests and EIA-positive tests over the study period. Using 28-d de-duplication (i.e., not counting repeat tests/positives within 28 d of an index test/positive as a new event). Figure B: Distribution of infectious and incubation periods for transmissions within 69 sequence types and 705 test CDI cases. All potential transmission links where the direction of transmission is known (ward contact after potential donor CDI and before recipient CDI) are plotted assuming maximum allowable infection and incubation periods of 26 wk and no ward contamination persisting after donor discharge (see Results for distributions from the most plausible links). Times are plotted rounded up to the nearest week, e.g., intervals of 0–6 d are plotted as 1 wk.
(DOC)

## Abbreviations

CDI: *Clostridium difficile* infection
EIA: enzyme immunoassay
MLST: multi-locus sequence typing
ORH: Oxford Radcliffe Hospitals
ST: sequence type
