# Supplementary material for: Characterisation of Clostridium difficile Hospital Ward–Based Transmission Using Extensive Epidemiological Data and Molecular Typing
Source: PLoS Med. 2012 Feb 7;9(2):e1001172. doi: 10.1371/journal.pmed.1001172 (PMC3274560; doi:10.1371/journal.pmed.1001172)
Supplement: Text S1 — Additional methods and results. Table A: Interventions in place at the start of the study (1 September 2007), and changes during the study (through 31 March 2010). Table B: Network characteristics. Figure A: C. difficile tests and EIA-positive tests over the study period. Using 28-d de-duplication (i.e., not counting repeat tests/positives within 28 d of an index test/positive as a new event). Figure B: Distribution of infectious and incubation periods for transmissions within 69 sequence types and 705 test CDI cases. All potential transmission links where the direction of transmission is known (ward contact after potential donor CDI and before recipient CDI) are plotted assuming maximum allowable infection and incubation periods of 26 wk and no ward contamination persisting after donor discharge (see Results for distributions from the most plausible links). Times are plotted rounded up to the nearest week, e.g., intervals of 0–6 d are plotted as 1 wk. (DOC) [file pmed.1001172.s001.doc]

**TEXT S1 – Additional Methods and Results**

**Computational Algorithm**

Ward contacts prior to a patient’s first EIA-positive culture-positive sample from September 2007 onwards were used to link each new CDI ‘test’ case (the ‘recipient’) to potential ‘donors’ with CDI of the same sequence type (ST) who had been present on the same ward at the same time as the ‘test’ case at some point in the past (Figure 1B). Contacts were defined to occur at a ward level as specific bed/bay information was not available electronically. Haemodialysis and chemotherapy were included as they are considered admissions in the NHS, but other outpatient attendances were not included.

For this analysis based on identifying the most plausible ST-restricted transmission links, we defined the ‘minimum infectious period’ as the time between the first EIA-positive culture-positive sample from the potential donor and the ward meeting of recipient and donor cases. We defined the ‘incubation period’ as the time between this meeting and the first EIA-positive culture-positive sample in the recipient case (Figure 1B).

Where the ward contact between two patients was greater than one day, the point of the most plausible potential transmission was chosen to minimize the infectious period, because donors were expected to be most infectious immediately following a EIA-positive stool sample after which they would have received presumptive treatment and been managed as if diarrhea was potentially infectious. Therefore where the donor sample was taken prior to the start of the ward contact, the point of transmission was assumed to be the start of the ward contact. Where the donor sample was taken during the contact, the point of transmission was assumed to be the date of the donor sample. Where donor-recipient pairs had more than one ward contact representing plausible transmissions, the contact that minimized the infectious period was chosen to define infection and incubation periods, as above.

In a second set of networks, we considered the possibility of transmission persisting after donor ward discharge. Where a potential donor left the ward before the recipient was admitted (ie no direct contact), we defined the ‘ward contamination time’ as the time between donor ward discharge and recipient ward admission, and the incubation period as the time from the recipient ward admission to EIA-positive sample. Where a donor was no longer considered infectious during an admission based on upper bounds on infection and incubation periods (see Results), ‘ward contamination’ could persist, such that transmission could occur to a recipient admitted shortly after the donor stopped being infectious.

Ward contacts indicated the direction of transmission from donor to recipient except where the two patients shared time and space on a ward before either was diagnosed with *C. difficile*. In this scenario, the direction of transmission was indeterminate and could represent a pre-symptomatic transmission event, with either patient considered a possible donor to the other.

We initially considered all possible ward contacts during the 26 weeks preceding each new CDI case (i.e. not longer than biologically expected[^1^](#_ENREF_6)). The donor-recipient pairs for each ST were represented as a network, with each case forming a node, linked to other nodes by possible transmission events with or without directionality as above. Groups of nodes joined by possible transmission events formed clusters. Within each ST, there could be single or multiple clusters – see Table S2 for a description of the network clusters. Within each cluster, the initial links made from ward contacts/contamination could contain biological inconsistencies, for example a recipient with more than one donor. To estimate the number of cases which could be linked via ward-based transmission, a pruning algorithm was applied in two stages. Firstly, starting with the earliest case in each cluster, directional links representing transmission to secondary cases were identified. Any cases linked to the earliest case by a link of indeterminate direction had this direction set to be consistent with transmission given the existing directional links. This provides an upper bound on the number of cases that can be plausibly linked together in each cluster. In the second stage, the network was examined for any biologically impossible link combinations, eg a recipient with two potential ‘donors’. Here, as above, only the link with the shortest infectious period was kept as this is most biologically plausible. The algorithm was repeated for each secondary case and each cluster until the entire network had been examined. For any remaining indeterminate links, the minimum infectious period was arbitrarily assumed to be the shorter of the two times from ward contact end to EIA-positive sample, and incubation period the longer. Thus any recipient linked to one or more ‘donors’ in the original network is always counted as a transmission link in the final network, but only the characteristics of the relationship with one donor (the most plausible assuming infectious period is minimized) is presented to avoid double counting. Table S2 also shows the characteristics of these most plausible (pruned) networks.

Several sensitivity analyses were conducted as described in the main Methods. Since the pruning algorithm could also affect the distribution of implied infection and incubation periods in the networks, these distributions were compared between networks both before and after pruning for main and sensitivity analyses, and were similar (eg see Figure 4 and Figure S1 for main analyses, data not shown for sensitivity analyses).

**Controls**

Clearly a proportion of contacts between patients may represent chance ward meetings without transmission occurring rather than transmission events. To calculate the proportion of links between cases that may be the result of chance meetings, the network analyses above were repeated using the same number of patients without *C. difficile* infection entered into a control network for each ST. The first groups of controls were EIA-negative patients, all of whom will have had diarrhea, and will therefore be more similar to CDI cases than general hospital controls which, if chosen at random will over-represent groups with lower CDI risks such as maternity cases. EIA-negative controls were matched on tests within ±3, ±15, or ±90 days of the EIA-positive case and for hospital exposure based on the number of unique hospital inpatients each case shared time and space with during the study. A second set of controls was drawn from all hospital inpatients, matched for hospital exposure, sex, age decade and admitted on the day of the case’s EIA-positive sample (or for cases testing EIA-positive in the community, matched for being admitted on the day the case was last discharged). Each set of controls was randomly drawn 1000 times, and the median proportion of controls linked by ward-based contact calculated. Net linked cases correcting the overall linked cases for chance ward meetings estimated from controls was estimated using the law of total probability, namely

P(link) = P(link|transmission)P(transmission) + P(link|no transmission)P(no transmission)

Assuming P(link|transmission)=1, estimating P(link|no transmission) from controls, and rearranging using P(no transmission)=1-P(transmission) gives

P(transmission) = (P(link)-P(link|no transmission))/(1-P(link|no transmission)

**Table A Interventions in place at the start of the study (September 2007), and changes during the study (through March 2010)**

| **Interventions in place at the start of the study (September 2007)** | | | |
| --- | --- | --- | --- |
| **Intervention** | | **Compliance monitoring** | |
| ***Antibiotic policy:*** Cephalosporin and fluroquinolone use restricted. Community acquired pneumonia: amoxicillin / co-amoxiclav ± macrolide; urinary tract infection: nitrofurantoin / co-amoxiclav; cellultis: flucloxacillin. Cephalosporin use in penicillin allergy, fluroquinolone for severe beta-lactam allergy. | | Pharmacy review of all inpatient prescriptions against hospital-wide antibiotic policy | |
| ***C difficile isolation policy:*** All patients with suspected C difficile infection, including patients with diarrhea of unknown cause (≥3 unformed stools in 24 hours) isolated in side rooms with en suite bathroom or dedicated commode wherever possible. Hand decontamination with soap and water, aprons and gloves worn for contact. Continued in confirmed cases until 48 hours following return to normal bowel habit. | | Monitored daily, Monday to Friday by infection control team review of all inpatients with diarrhea. Frequency of monitoring decreased to 3 times a week from 2008 to 2009, and once a week during 2010. Spot checks of patient notes undertaken to ensure infection control service aware of all patients with suspected *C difficile*, confirmed cases notified by laboratory to infection control team. | |
| ***Cleaning:*** Actichor plus combined detergent and Sodium hypochlorite solution (called an “enhanced clean”) used for once daily clean of every surface and wall in room with patient with suspected *C difficile*. | | Daily monitoring, Monday to Friday of the number of additional enhanced cleans requested with the number of patients with suspected or confirmed active *C difficile* cases. Records from procurement monitored Monday to Friday. Frequency reduced over time as above. | |
| ***C difficile testing:*** Three samples to be sent for C difficile testing from any patients with suspected infectious diarrhea (≥3 unformed stools in 24 hours) | | Reviewed as part of infection control monitoring of these patients as above | |
| ***Mandatory C difficile testing:*** Mandatory testing of all diarrheal samples sent from patients aged 65 years or older for *C. difficile*, regardless of whether test requested | | Number of EIA tests increases from 400-500 per month before, to 800-1000 per month after introduction in May 2007 | |
| ***C difficile treatment:*** Empirical treatment of oral vancomycin 125mg qds, continued for 14 days if diagnosis confirmed | | Initiation of treatment confirmed by infection control team for all confirmed cases. Pharmacy monitoring of duration of treatment. | |
| ***Feedback:*** Failure to comply with infection control policy on isolation and cleaning escalated to ward and then senior management until compliance achieved. | |  | |
| Study starts (September 2007) | | | |
| **Date of Change** | **Intervention** | | **Compliance Monitoring** |
| **January 2009** | ***Care pathway:*** *C. difficile* care pathway added to policy  Care checklist including communication of diagnosis to patient, family, and medical team; review of medication (antibiotics, acid suppression, laxatives); fluid balance monitoring (fluid and stool chart); nutritional assessment; skin integrity assessment; sepsis monitoring; compliance with isolation as above | | All confirmed *C difficile* cases reviewed by infection control team to ensure care pathway in place |
| **September 2009** | ***C. difficile isolation policy:*** Isolation as previously, but now within 2hrs of suspicion  Exception where patients requiring one-to-one monitoring or care and unsuitable for side room care | | Monitored Monday to Friday by infection control service at introduction, then 3 times a week and then weekly as above |
| **December 2009** | ***Sending of stool specimens:*** Reduction in number of stool samples tested. Sending one diarrheal specimen and if negative then send a second | | Monitored weekly from January 2010, over testing reported back weekly to wards |

**Table B** Network characteristics

|  | **Maximum allowable infectious and incubation periods 26 weeks** | | | | **Maximum allowable infectious and incubation periods 8 and 12 weeks respectively** | | | |
| --- | --- | --- | --- | --- | --- | --- | --- | --- |
|  | **Most plausible links** | | **All possible links** | | **Most plausible links** | | **All links** | |
| **Cluster size** | **Cluster frequency** | **Test cases in clusters (%)** | **Cluster frequency** | **Test cases in clusters (%)** | **Cluster frequency** | **Test cases in clusters (%)** | **Cluster frequency** | **Test cases in clusters (%)** |
| **1** | 408 | 408 (58%) | 403 | 403 (55%) | 465 | 465 (66%) | 463 | 463 (66%) |
| **2** | 44 | 85 (12%) | 37 | 71 (10%) | 46 | 90 (13%) | 44 | 86 (12%) |
| **3** | 21 | 58 (8%) | 16 | 43 (6%) | 22 | 62 (9%) | 19 | 53 (8%) |
| **4** | 8 | 31 (4%) | 8 | 29 (4%) | 6 | 24 (3%) | 4 | 16 (2%) |
| **5** | 3 | 15 (2%) | 2 | 10 (1%) | 1 | 5 (1%) | 2 | 10 (1%) |
| ≥**6** | 10 | 108 (15%) | 8 | 149 (21%) | 7 | 59 (8%) | 2 | 77 (11%) |
| **Cases with a credible donor** |  | 223/705 (32%) |  |  |  | 165/705 (23%) |  |  |

Note: clusters are included where one or more cases in a cluster fell within the ‘test’ period (March 2008-March 2010). 'Test' cases may be present in a cluster as a donor, recipient or both.

**Figure A C. difficile tests and EIA-positive tests over the study period**

**Figure B Distribution of infectious and incubation periods for transmissions within 69 sequence types and 705 ‘test’ CDI cases**

**References**

1. Cohen SH, Gerding DN, Johnson S, et al. Clinical practice guidelines for Clostridium difficile infection in adults: 2010 update by the society for healthcare epidemiology of America (SHEA) and the infectious diseases society of America (IDSA). *Infect Control Hosp Epidemiol.* May 2010;31(5):431-455.
